# Effect of ocular demodicosis on the stability of the tear film and the tear break up time

**DOI:** 10.1038/s41598-021-03801-y

**Published:** 2021-12-21

**Authors:** Aleksandra Sędzikowska, Witold Tarkowski, Joanna Moneta-Wielgoś, Krzysztof Grzyliński, Grzegorz Tarkowski, Daniel Młocicki

**Affiliations:** 1 Department of General Biology and Parasitology, Medical University of Warsaw, 02-004 Warsaw, Poland; 2 Tarnow Optical Center, Wałowa 8 Street, 33-100, Tarnów, Poland; 3 TG Pharma, Piłsudskiego 2 Square, 00-073 Warsaw, Poland; 4 Department of Ophthalmology, Military Institute of Medicine, Warsaw, Poland; 5 Kazimierz Pulaski University of Technology and Humanities, Radom, Poland; 6 W. Stefański Institute of Parasitology, Polish Academy of Sciences, Twarda 51/55 Street, 00-818 Warsaw, Poland

**Keywords:** Microbiology, Diseases, Medical research

## Abstract

The aim of the study was to analyze the correlation between the presence of *Demodex* mites in the hair follicles of patients' eyelashes and the stability and break up time of the tear film assessed with the Non-Invasive Tear Break Up Times (NIBUT) method. 319 patients were included in the study (195 women, 124 men). The patients were divided into two groups: those with *Demodex* infestation and without visible symptoms of eyelid or eye surface diseases, and asymptomatic non-infested patients. The NIBUT analysis was performed with a 5 M keratograph (oculus). Non-invasive tests were performed to identify the first and mean values of the tear break up time. The first and mean tear break up time in the *Demodex*-infested group was lower than in the non-infested subjects. The difference was a highly statistically significant. There was a significant correlation with the age of the patients for the first break up time. The first break up time in both eyes decreased with the age of the *Demodex*-infested and non-infested patients. The NIBUT analyses indicate the impact of *Demodex* mites on the tear film stability. This may suggest possible association of demodicosis with dry eye syndrome.

## Introduction

**Figure 1:**
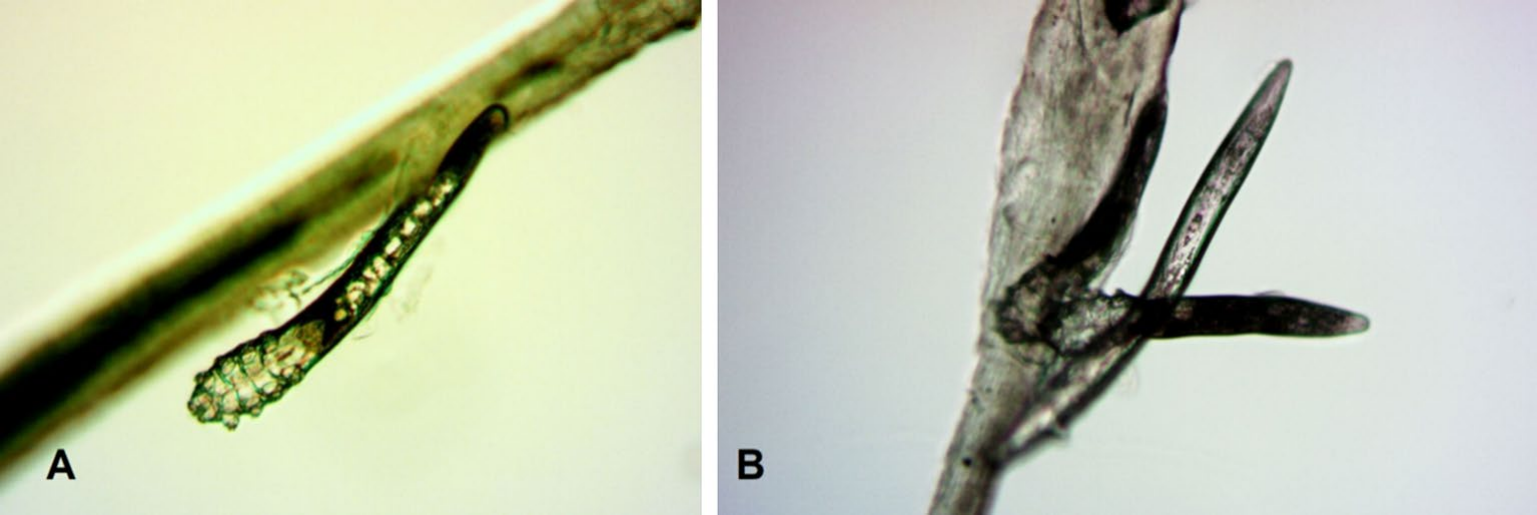
Adult stage of *Demodex folliculorum* **(A)** and two adults and one larva of *D. folliculorum* **(B)**. Photo: Witold Tarkowski.

*Demodex* are a small mites with high host specificity found in many different species of mammals, including humans. To date, two species have been described in humans: *Demodex brevis* and *Demodex folliculorum* (Fig. 1), which colonize respectively hair follicles or sebaceous glands. Numerous studies indicate that infestation with these parasites may play a role in eyelid margin inflammation, chalazion, pterygium, rosacea, acne vulgaris, and seborrheic dermatitis^1–5^ or may prompt abandonment of contact lenses, including therapeutic lenses, by their users^6^.

The tear film is a layer covering and protecting the eye surface from the external environment. It is generally considered to consist of three layers: the outermost lipid layer, the middle aqueous layer, and the innermost mucin layer adjacent to the ocular surface. Its thickness ranges from 7 to 10 µm and the time of its persistence on the globe is from 12 to 15 s^7,8^. The lipid layer is produced by Meibomian glands located in the tarsal plate of upper and lower eyelids. It inhibits evaporation of the underlying aqueous layer, ensures a smooth and regular optical surface of the cornea, produces surface tension of the tear film, preventing tears from flowing along the eyelid margin, and lubricates the eyelids thus reducing friction during their movement over the ocular surface^9^. The aqueous layer is produced by lacrimal glands, which secrete not only water but also various proteins and electrolytes. This layer delivers oxygen and nutrients to the underlying cornea and flushes away epithelial debris, toxins, and foreign bodies^10^. Due to the presence of immunoglobulins, lysozyme, and lactoferrin, it serves an antibacterial function as well^11^. The mucin layer consists mainly of glycoproteins secreted by conjunctival goblet cells and forming the glycocalyx, i.e. a protein mesh converting the hydrophobic surface of the corneal epithelium into a hydrophilic one^9^. Mucin elements disperse in the aqueous layer and their concentration increases towards the eye surface^12^.

The lipid layer of the tear film mainly comes from the Meibomian glands and spreads with blinking^9,13^. The eyelid muscles while blinking also stimulates the secretion of Meibomian lipids, which moisturize the surface of the eye and inhibit the evaporation of tears^14^. The most important factor involved in the rapid spread and stability of the tear film is the interaction of glycocalyx with water^11^.

The tear film can be influenced by many factors, e.g. blinking, lacrimal apparatus, ambient conditions (temperature, humidity), and emotions^15^. Its instability may result in dry-eye syndrome, which is one of the most commonly reported ocular ailments^16^. According to TFOS-DEWS II (Dry Eye Workshop)^17^, dry-eye syndrome is a multifactorial pathology of tears and the ocular surface with such manifestations as discomfort, visual disturbances, and instability of the tear film increasing the risk of damage to the ocular surface, which is accompanied by hyperosmolarity of the tear film and inflammation on the ocular surface. Sidique and Braun^18^ examined how well the floating lipid layer can be approximated by a insoluble surfactant monolayer in the context of lubrication theory. Their model includes the effects of surface tension, insoluble surfactant monolayer transport, solutal Marangoni effects, evaporation, osmolarity transport, osmosis and wettability of corneal surface. According to these observations the solutal Marangoni effect, for local increases in surfactant concentration, can induce local thinning and this effect seems to dominate the reduction in thinning rate due to evaporation. Their noted that osmolarity in the tear film increases because water lost to the average evaporation rate and to a lesser extent by flow inside the film. This model predicts that the Marangoni effect coupled with evaporation can determine the location of first breakup; it also agrees with other models of breakup that predicts elevated osmolarity when breakup occurs^18^.

**Figure 2:**
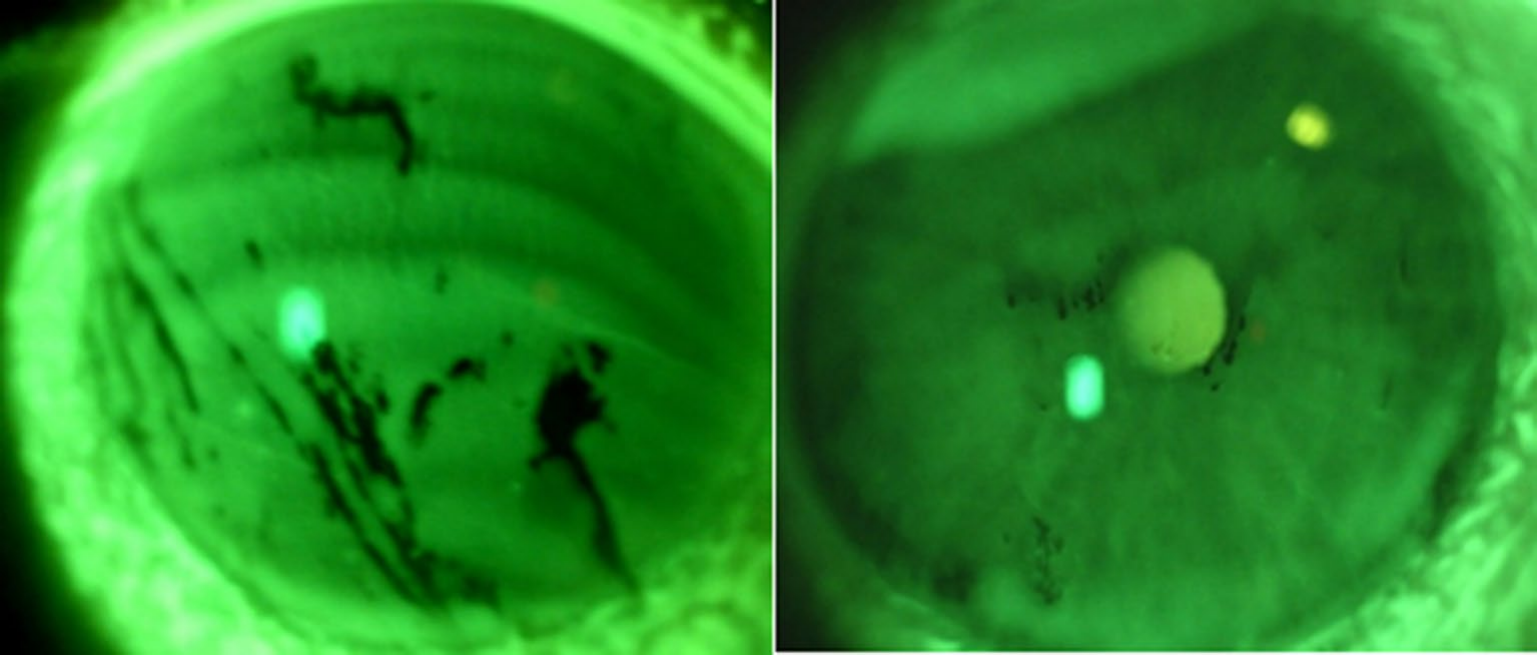
TBUT test using a slit lamp by a wide open slit in blue light (unpublished picture taken by Witold Tarkowski.

The basic method for assessment of the stability of the precorneal tear film is the tear film break up time test (TBUT). To perform this test, fluorescein is instilled into the lower fornix and the subject is asked to blink a few times and then to stop blinking. TBUT is assessed using a slit lamp by a wide open slit in blue light; after some time, appearing black spots or lines appear indicate dry areas of the cornea (Fig. 2)^19,20^.

The TBUT/NIBUT and osmolarity of the tear film are two parameters that change in each type of dry-eye syndrome. For this reason, the tear break up time is one of the basic tests in examining patients with this syndrome. Break up time examinations can also be performed with a non-invasive method using keratometers, computerized systems, and topographic analysis systems, e.g. videokeratoscopy, ocular surface thermography, or lateral shearing interferometry^21,22^. These devices eliminate reflex tearing associated with the administration of fluorescein to the conjunctival sac and thus considerably increase the specificity of the test.

The aim of the study was to analyze the correlation between the presence of *Demodex* mites in the hair follicles of asymptomatic patients' eyelashes and the stability and break up time assessed with the NIBUT method.

## Materials and methods

All experimental protocols were approved by Bioethics Committee of the Medical University of Warsaw. Consent from the Bioethics Committee of the Medical University of Warsaw was obtained prior to the research (consent number: KB/240/2015). Patients classified into the study were carefully examined by ophthalmologist using the slit lamp to assess the status of eyelids and ocular surface. The patients with blepharitis, pterygium, chalazion, conjunctivitis etc. were excluded from the study. In total, 319 patients visiting ophthalmologist were included into the study and underwent the non-invasive break up time (NIBUT) test. Informed consent was obtained from all participants. The patients were divided into two groups: those with and without *Demodex* infestation (Table 1).

**Table 1:** Characteristics of the groups.

		*Demodex* positive n = 157	*Demodex* negative n = 162	p
Sex, n (%)	Female	108 (68,8%)	87 (53,7%)	0.0057^(1)^
	Male	49 (31,2%)	75 (46,3%)	
Age (years)	M ± SD	51,7 ± 18,3	37,8 ± 13,5	< 0.0001^(2)^
	Median (min.–max.)	48,0 (22–93)	34,5 (14–83)	

Arithmetic mean (M), standard deviation (SD), p-value (p).

^(1^^)^Pearson's chi-square test result, ^(2)^t-test result with Cochran–Cox correction.

Material for studying the presence of *Demodex* were eyelashes collected from all patient,. (7–10 eyelashes), by means of sterile tweezers. The eyelashes were placed on a glass slide and soaked with a drop of Hoyer's solution^21,22^ (40 cm^3^ distilled water, 24 g gum arabic, 160 g chloral hydrate, 16 g glycerine). The specimens were observed under an light microscope under 200–400 × magnification. A positive result was recorded where adult, larvae, nymphs or eggs of *Demodex* sp. were detected in the material.

### Analysis of non-invasive break up time (NIBUT)

**Figure 3:**
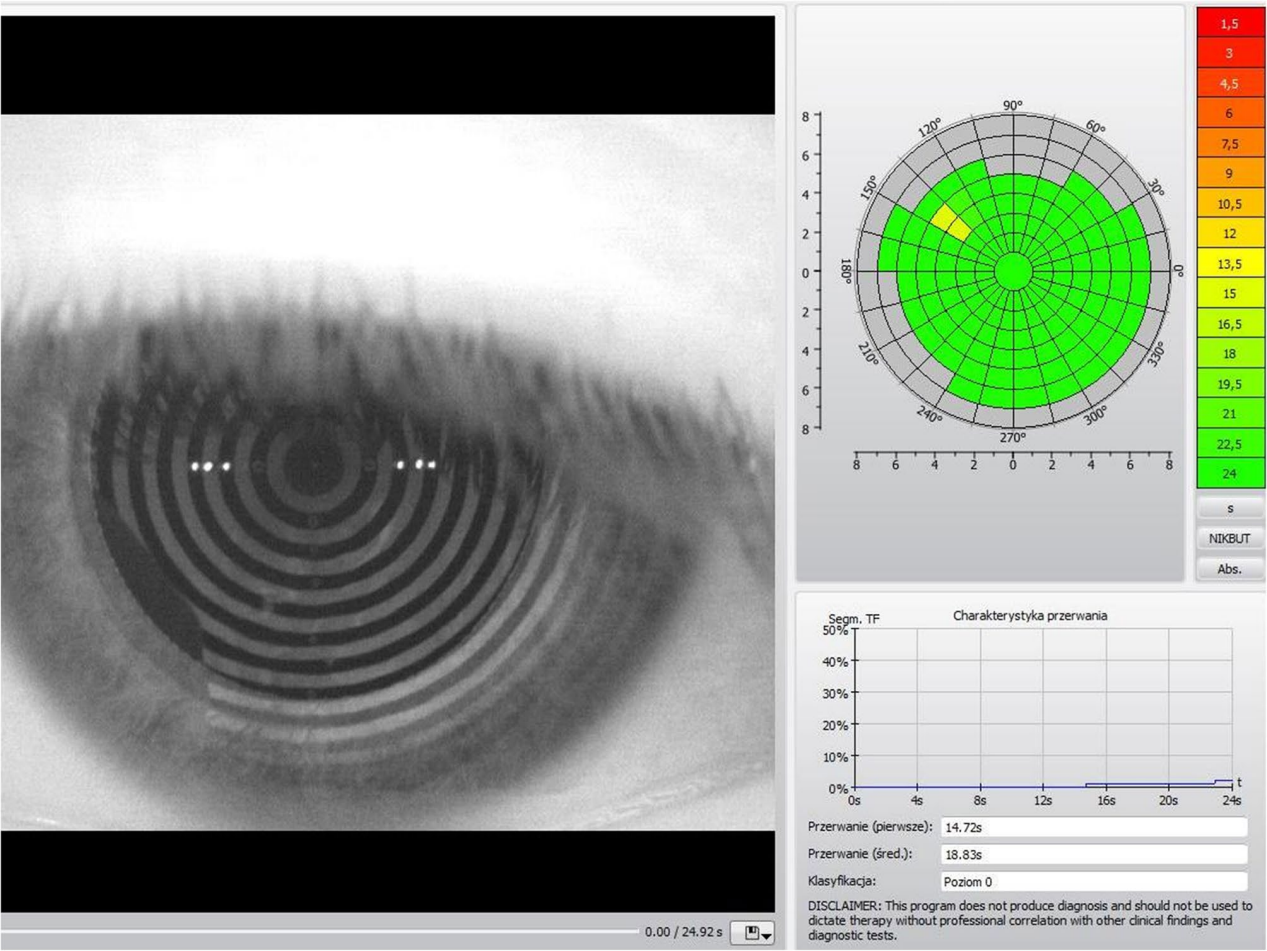
The TearMap by Keratograph 5 M: the respective break up time is graphically illustrated for each segment in seconds—stable tear film (green segments indicate the areas of the stable tear film). Photo: Witold Tarkowski.

**Figure 4:**
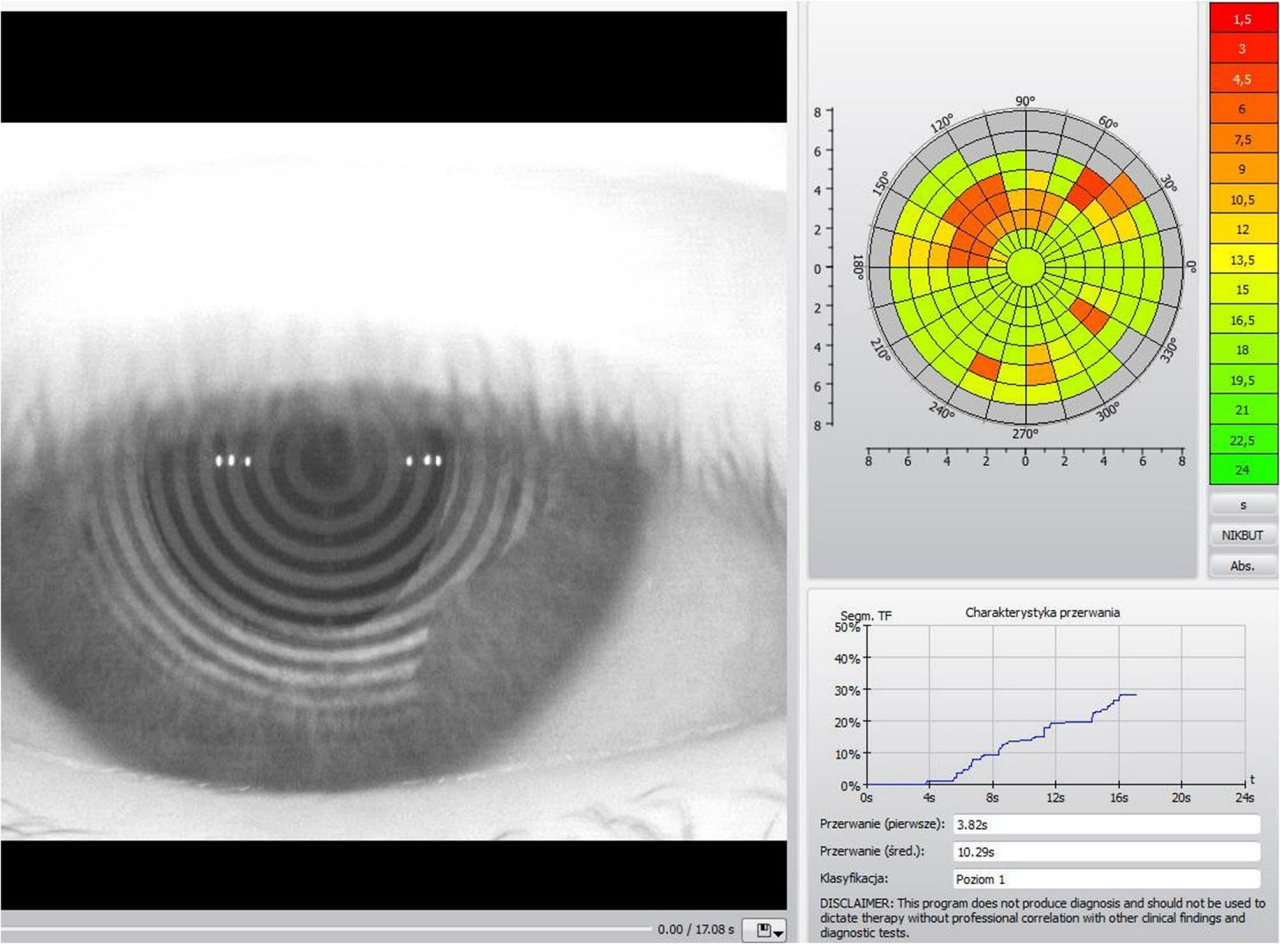
The TearMap by Keratograph 5 M: the respective break up time is graphically illustrated for each segment in seconds—unstable tear film (red and orange segments are related to unstable tear film). Photo: Witold Tarkowski.

The non-invasive break up time analysis was performed with a 5 M keratograph (Oculus) with software for infrared light NIBUT examination. All methods were carried out in accordance with relevant guidelines and regulations. The measured break up time is evaluated digitally with simultaneous color-coding. A grid is used to divide the surface of the examined cornea into segments. Red areas signify an “unstable tear film” and green areas indicate a “stable tear film” (Figs. 3, 4).

The first break-up time is the shortest time to the occurrence of the process in the first segment. The mean break-up time is calculated as an arithmetic mean of the break up times in all segments up to a maximum of 24 s of the examination time. The first and mean break up times in all patients were archived in the device's memory. The first break up time of less than 10 s was considered abnormal^19^.

### Statistical analysis

The first and mean non-invasive break up time measurements in the healthy subjects and the *Demodex-*infested patients were analyzed statistically. A value of p < 0.05 was considered statistically significant. Statistical calculations were performed using the STATISTICA 10 PL statistical package. The t-test was used to check the significance of the differences in the tear break up time in the group of the *Demodex*-infested and non-infested patients. When the condition of homogeneity of variance was not met, the t-test with Cochran–Cox correction was applied. The Pearson linear correlation coefficient significance test was used to determine the correlation between the age of the patients and the tear break-up time.

## Results

To determine the possible effect of *Demodex* on the tear break up time, 319 patients were examined. Non-invasive tests were performed to identify the first and mean values of the tear break up time. The examined group included asymptomatic patients presenting with *Demodex* infestation and non-infested subjects.

The t-test with Cochran-Cox correction showed a statistically significant difference in the first tear break up time between the *Demodex*-infested patients and the non-infested subjects in the right (Table 2) and left eye (Table 3). The mean first tear break up time in the right eye was lower in the *Demodex*-infested group (8.00 s) than in the non-infested subjects (14.73 s). The same result was noted in the case of the left eye, i.e. a shorter time was observed in the *Demodex*-infested patients (8.95 s) in comparison with the healthy subjects (14.80 s). The mean results from both eyes are statistically significant as well and show a significantly shorter time in the *Demodex*-infested group than in the other subjects (Table 4).

**Table 2:** Descriptive statistics of the first break up time in the right eye in patients with and without *Demodex* infestation and results of the t-test with Cochran–Cox correction.

Group	n	NIBUT (seconds) First break up time, right eye					t	df	p
		M ± SD	Variance	Median	Q25–Q75	Min–max			
*Demodex* positive	157	8.00 ± 5.47	29.98	6.37	4.21–10.32	1.91–24.09	-9.65	304.9	< 0.0001
*Demodex* negative	162	14.73 ± 6.92	47.89	13.87	8.60–22.18	2.29–24.00			

Arithmetic mean (M), standard deviation (SD), interquantile range (Q25–Q75), degrees of freedom (df), t-value (t), p-value (p).

**Table 3:** Descriptive statistics of the first break up time in the left eye in patients with and without *Demodex* infestation and results of the t-test with Cochran–Cox correction.

Group	N	NIBUT (seconds) First break up time, left eye					t	df	p
		M ± SD	Variance	Median	Q25–Q75	Min–max			
*Demodex* positive	157	8.95 ± 6.11	37.29	7.14	4.46–10.71	1.53–24.48	−8.08	315.2	< 0.0001
*Demodex* negative	162	14.80 ± 6.80	46.18	13.38	8.99–22.94	2.68–24.09			

Arithmetic mean (M), standard deviation (SD), interquantile Range (Q25–Q75), degrees of freedom (df), t-value (t), p-value (p).

**Table 4:** Descriptive statistics of the first break up time in both eyes in patients with and without *Demodex* infestation and results of the t-test with Cochran–Cox correction.

Group	n	NIBUT (seconds) First break up time, both eyes					t	df	p
		M ± SD	Variance	Median	Q25–Q75	Min–max			
*Demodex* positive	157	8.48 ± 4.36	19.03	7.46	4.78–11.57	1.91–24.00	−11.63	309.7	< 0.0001
*Demodex* negative	162	14.76 ± 5.26	27.64	14.48	10.71–17.79	4.21–24.00			

Arithmetic mean (M), standard deviation (SD), interquantile range (Q25–Q75), degrees of freedom (df), t-value (t), p-value (p).

The t-test showed a statistically significant difference in the mean tear break up time in the right eye (Table 5) and the left eye (Table 6) between the *Demodex*-infested and non-infested patients. The mean tear break up time in the right eye was lower in the *Demodex*-infested group (12.08 s) than in the non-infested subjects (17.89 s). In turn, the break up time in the left eye was 12.86 s and 18.15 s, respectively. The mean values from both eyes were statistically significant as well and showed a shorter tear break up time in both eyes of the *Demodex*-infested patients than in the non-infested subjects (Table 7).

**Table 5:** Descriptive statistics of the mean break up time in the right eye in patients with and without *Demodex* infestation and results of the t-test with Cochran–Cox correction.

Group	n	NIBUT (seconds) Mean break up time, right eye					t	df	p
		M ± SD	Variance	Median	Q25–Q75	Min–max			
*Demodex* positive	157	12.08 ± 5.47	29.88	11.09	7.95–15.76	2.50–24.09	−9.92	317	< 0.0001
*Demodex* negative	162	17.89 ± 4.99	24.89	18.64	13.96–22.69	7.00–24.00			

Arithmetic mean (M), standard deviation (SD), interquantile range (Q25–Q75), degrees of freedom (df), t-value (t), p-value (p).

**Table 6:** Descriptive statistics of the mean break up time in the left eye in patients with and without *Demodex* infestation and results of the t-test with Cochran-Cox correction.

Group	N	NIBUT (seconds) Mean break up time, left eye					t	df	p
		M ± SD	Variance	Median	Q25–Q75	Min–max			
*Demodex* positive	157	12.86 ± 5.91	34.98	11.79	8.47–17.70	1.53–24.00	−8.85	296.6	< 0.0001
*Demodex* negative	162	18.15 ± 4.67	21.81	18.35	15.02–23.17	6.19–24.09			

Arithmetic mean (M), standard deviation (SD), interquantile range (Q25–Q75), degrees of freedom (df), t-value (t), p-value (p).

**Table 7:** Descriptive statistics of the mean break up time in both eyes in patients with and without *Demodex* infestation and results of the t-test with Cochran–Cox correction.

Group	N	NIBUT (seconds) Mean break up time, both eyes					t	df	p
		M ± SD	Variance	Median	Q25–Q75	Min–max			
*Demodex* positive	157	12.47 ± 4.94	24.38	11.92	8.44–16.59	2.02–24.00	−11.23	292.8	< 0.0001
*Demodex* negative	162	18.02 ± 3.80	14.41	18.15	15.73–20.47	7.23–24.00			

Arithmetic mean (M), standard deviation (SD), interquantile range (Q25–Q75), degrees of freedom (df), t-value (t), p-value (p).

### AGE and NIBUT

#### *Demodex*-infested patients

The analysis of the Pearson linear correlation coefficient showed a significant correlation with the age of the *Demodex*-infested patients only for the first break up time (p = 0.0026). The correlation was negative and weak (r = −0.239). The first break up time in both eyes decreased with the age of the *Demodex*-infested patients (Table 8).

**Table 8:** Results of the significance the Pearson linear correlation between the age of the *Demodex*-infested patients and the first and mean break up times in both eyes.

*Demodex*-infested patients				
A pair of variables	n	r	t	p
Age (years) & first break up time	157	−0.239	−3.07	**0.0026**
Age (years) & mean break up time	157	−0.141	−1.77	0.0788

Pearson's linear correlation coefficient (r), t-value (t), p-value (p).

Significant value in bold.

#### Non-infested patients

The analysis of the Pearson linear correlation coefficient revealed a significant correlation with the age of the non-infested patients only for the first break up time (p = 0.0485). The correlation was negative and very weak (r = −0.155). The first break up time in both eyes decreased with the age of the non-infested patients (Table 9).

**Table 9:** Results of the significance the Pearson linear correlation between the age of the non-infested patients and the first and mean break-up times in both eyes.

*Demodex* non-infested patients				
A pair of variables	n	r	t	p
Age (years) & first break up time	162	−0.155	−1.99	**0.0485**
Age (years) & mean break up time	162	−0.081	−1.03	0.3068

Pearson's linear correlation coefficient (r), t-value (t), p-value (p).

Significant value in bold.

## Discussion

The present study demonstrated a difference in the tear break up time between the patients with diagnosed demodicosis without visible eye lid or eye surface disorders, and the non-infested subjects. As indicated in our study the first tear break up time was significantly reduced in the *Demodex*-infested group in comparison with the non-infested subjects. The same trend was observed in the mean tear break up time. Other researchers observed significantly shorter TBUT in *Demodex* positive patients that underwent routine cataract surgery^23^. Rabensteiner et al.^24^ determined the tear break up time with the use of fluorescein, which was shorter in a *Demodex*-infested group (3.35 s) than in non-infested patients (3.7 s). However, these results were statistically insignificant and were obtained with an invasive TBUT method. It is worth emphasizing that the fluorescein test is burdened with error resulting from its invasiveness and the lower accuracy of measurement of time. However, some authors compare NIBUT Assessed With Video-Corneal Topography to the Standard Invasive TBUT suggesting that non-invasive and invasive methods are comparable^25^.

Our results indicate a significant correlation between the presence of *Demodex* mites in the eyelash hair follicles and the reduction of non-invasive tear break-up time, and thus lower stability of the tear film. This indicates the usefulness of non-invasive measurement techniques to analyze the stability and tear break-up time in patients with *Demodex* infestation. The tear break-up time test is one of the basic examinations of patients with dry eye syndrome^26^. As specified by the definition, the syndrome is a “disorder of the tear film due to tear deficiency or excessive evaporation, which causes damage to the interpalpebral ocular surface and is associated with symptoms of ocular discomfort”^13^. The definition of dry-eye syndrome was expanded in the following years, and the proposed criteria for diagnosis may differ between some countries^27,28^. The glycocalyx is very delicate and could be damage very easily^29^. There is possibility that the debris from *Demodex* lead to bits of the glycocalix breaking off and then the surface is less wettable^30,31^.

Meibomian gland dysfunction (MGD) causes disturbances in the composition or reduces the amount of the lipid secretion in the tear film, which leads to increased evaporation and reduced tear stability as well as loss of eye surface lubrication, followed by damage to the ocular surface epithelium^32^. As reported by Schachter et al.^33^, the presence of *Demodex* mites may be associated with MGD. Although *Demodex* infestation is diagnosed in a large part of the population^34–36^, it is not entirely clear whether the tear break-up time is reduced in all infected patients. Cheng et al.^37^ suggests that the greater the number of *Demodex* mites, the more severe the damage to Meibomian glands. Concurrently, other organisms present on the eyelid, e.g. *Staphylococcus aureus* bacteria, can exert an effect on the functioning of the eye surface. These bacteria decompose fats and esters, and the metabolites of these processes can diffuse through the aqueous layer into the mucin layer, resulting in its hydrophobicity, which in turn leads to instability of the tear film. It has been found that patients with *Demodex* blepharitis have varying degrees of imbalance of bacterial microbiota in the conjunctival sac^38^. In addition, the number of *Demodex* mites affects specific bacteria on the surface of the eye, manifested by discomfort in the eye and obvious symptoms of blepharitis. It is probable that *Demodex* infestations can disrupt the eye microbiome and thus contribute to tear film instability^32^. Nevertheless, further detailed analyses of the association of demodicosis with changes in the eye microbiome are required. Tear film instability of may be also related to debris from *Demodex* that may lower surface tension leading and subsequent reduction in tear film thickness, as indicated for other impurities^13^.

Moreover, the inflammatory processes of Meibomian glands and eye surface may be influenced by *Demodex* infestation^39^. It has been suggested that *Demodex* mites may play an aggravating role in inflammatory ocular surface disorders^40^.

Owczyńska et al.^41^ reported a positive correlation between the presence of *Demodex* mites and the discomfort of work in front of the computer screen resulting from destabilization of the tear film manifested by shortened NIBUT. The percentage of surveyed office staff working over 4 h a day with a computer who reported such symptoms as burning, itching, gritty eyes, dry eyes, and periodic excessive tearing combined with reddened eyes and were diagnosed with demodicosis was 85.71%. In turn, only 20.83% of *Demodex*-infested respondents did not report discomfort.

The search for factors that may affect tear film stability is an important line of research . Lee et al.^42^ indicated a strong correlation between the number of *Demodex* and the severity of ocular discomfort, suggesting that *Demodex* plays a pathogenic role in the ocular discomfort linked with aging. Our research shows that the presence of *Demodex* mites on the eyelids may lead to disturbances in the tear film stability. To discover the cause of this problem, the research should be continued and include the determination of the osmolarity, tear film composition, microbiome structure, and eyes immunology of the tear film in *Demodex*-infested patients.

## Conclusions

Tear film instability may lead to discomfort and contributing to the development of ocular surface diseases and corneal damages. Various causes of the reduction of tear break-up time and instability of the tear film have been suggested. The present non-invasive tear break-up time (NIBUT) analyses in patients with and without *Demodex* clearly indicate that the infestation influences the first and mean tear film break-up time. Concurrently, attention should be paid to the possible impact of *Demodex* presence on the physicochemical tear film parameters and structure of the eye microbiome. Therefore, in future, comprehensive studies of the tear film composition in *Demodex*-infested patients should be conducted and the influence of these mites on the eye surface immunology, tear film composition and thickness, and changes in the eye surface microbiome structure should be assessed.
